# A para-aortic malignant melanotic nerve sheath tumor mimicking a gastrointestinal stromal tumor: a rare case report and review of literature

**DOI:** 10.1186/s12893-022-01727-4

**Published:** 2022-07-28

**Authors:** Kuan-Yu Lin, Lujen Chen, Siu-Wan Hung, Sheng-Chun Hung, Cheng-Kuang Yang, Chih-Jung Chen, Kun-Yuan Chiu

**Affiliations:** 1 School of Medicine, Chung Shan Medical University, Taichung, Taiwan, R.O.C.; 2 Department of Pathology and Laboratory Medicine, Taichung Veternas General Hospital, Taichung, Taiwan; 3 Interventional Radiology, Radiology Department, Taichung Veterans General Hospital, Taichung, Taiwan, R.O.C.; 4 School of Medical Imaging and Radiological Sciences, Chung Shan Medical University, Taichung, Taiwan, R.O.C.; 5 Division of Urology, Department of Surgery, Taichung Veterans General Hospital, Taichung, Taiwan, R.O.C.; 6 Institute of Medicine, Chung Shan Medical University, Taichung, Taiwan, R.O.C.; 7 Department of Applied Chemistry, National Chi Nan University, Nantou, Taiwan, R.O.C.

**Keywords:** Malignant melanotic nerve sheath tumor, Melanotic schwannoma, Gastrointestinal stromal tumor, Abdominal cavity, Case report

## Abstract

**Background:**

Malignant melanotic nerve sheath tumor (MMNST), formerly called melanotic schwannoma, is a rare tumor of neural crest derivation which most frequently arises from the region of spinal or autonomic nerves near the midline. Recent studies have reported malignant behavior of MMNST, and there still has no standard management guidelines. Intra-abdominal MMNST, which has never been reviewed as an entity, is even rarer. In this study, we present a rare case of a cystic MMNST arising from the para-aortic region and mimicking an intra-abdominal gastrointestinal stromal tumor (GIST), and review the literature regarding MMNSTs located in the abdominal cavity.

**Case presentation:**

A 59-year-old female was incidentally found a tumor located in the left para-aortic area by non-contrast computed tomography. A Magnetic Resonance Imaging showed a cystic mass originated from the inferior mesenteric artery (IMA) territory. A GIST was initially diagnosed. The tumor was resected en bloc by laparoscopic surgery and was found between mesocolon and Gerota’s fascia with blood supply of IMA. Grossly, dark brown materials were noted at the inner surface of the cystic wall. Microscopically, the tumor cells were melanin-containing, and no psammomatous bodies were present. Immunohistochemically, the tumor showed positivity for MART1, HMB45, collagen IV, and SOX10, and negativity for AE1/AE3. MMNST was favored over malignant melanoma, since the tumor was located near ganglia and had cells with less atypical cytology and a low mitotic rate, and subsequent adjuvant radiotherapy was performed. The patient was alive with no evidence of recurrent or metastatic disease 11 months after radiotherapy.

**Conclusions:**

Our review of abdominal MMNST cases showed a female predominance, with an average age of 54.8 years, and a trend toward being a larger tumor showing cystic or necrotic changes. Local recurrence and metastasis rate were reviewed, and both showed a low rate. Diagnosis of MMNST should combine all the available findings, and complete excision of the tumor should be performed, followed by long-term patient monitoring.

## Background

Malignant melanotic nerve sheath tumor (MMNST), also called melanotic schwannoma, is a rare tumor of neural crest derivation composed of variably melanin-producing Schwann cells [1, 2]. MMNST, first recorded by Millar in 1932 [3], most frequently arises from the region of spinal or autonomic nerves near the midline [1]. MMNST has been reported to be associated with Carney’s complex (skin pigmentary abnormalities, myxomas, endocrine tumors, or endocrine overactivity) [2, 4, 5]. Most melanotic schwannomas are clinically benign tumors [4]. However, recent studies have reported malignant behavior of tumors that show local recurrence and metastasis [2, 6].

MMNST still has no standard treatment guidelines, and complete excision of the tumor is the most common approach. In the present study, we present a rare case of a cystic MMNST arising from the para-aortic region and mimicking an intra-abdominal gastrointestinal stromal tumor (GIST). We also discuss the radiologic and histologic features, and the literature regarding MMNSTs located in the abdominal cavity is reviewed.

## Case presentation

A 59-year-old female was referred for a tumor located in the left para-aortic area, close to the pancreatic tail and left renal hilum, found incidentally by non-contrast computed tomography (CT) during a urolithiasis survey. This patient had a past history of hypertension, but it was under control. She was asymptomatic, without any abnormal physical examination findings.

**Fig. 1:**
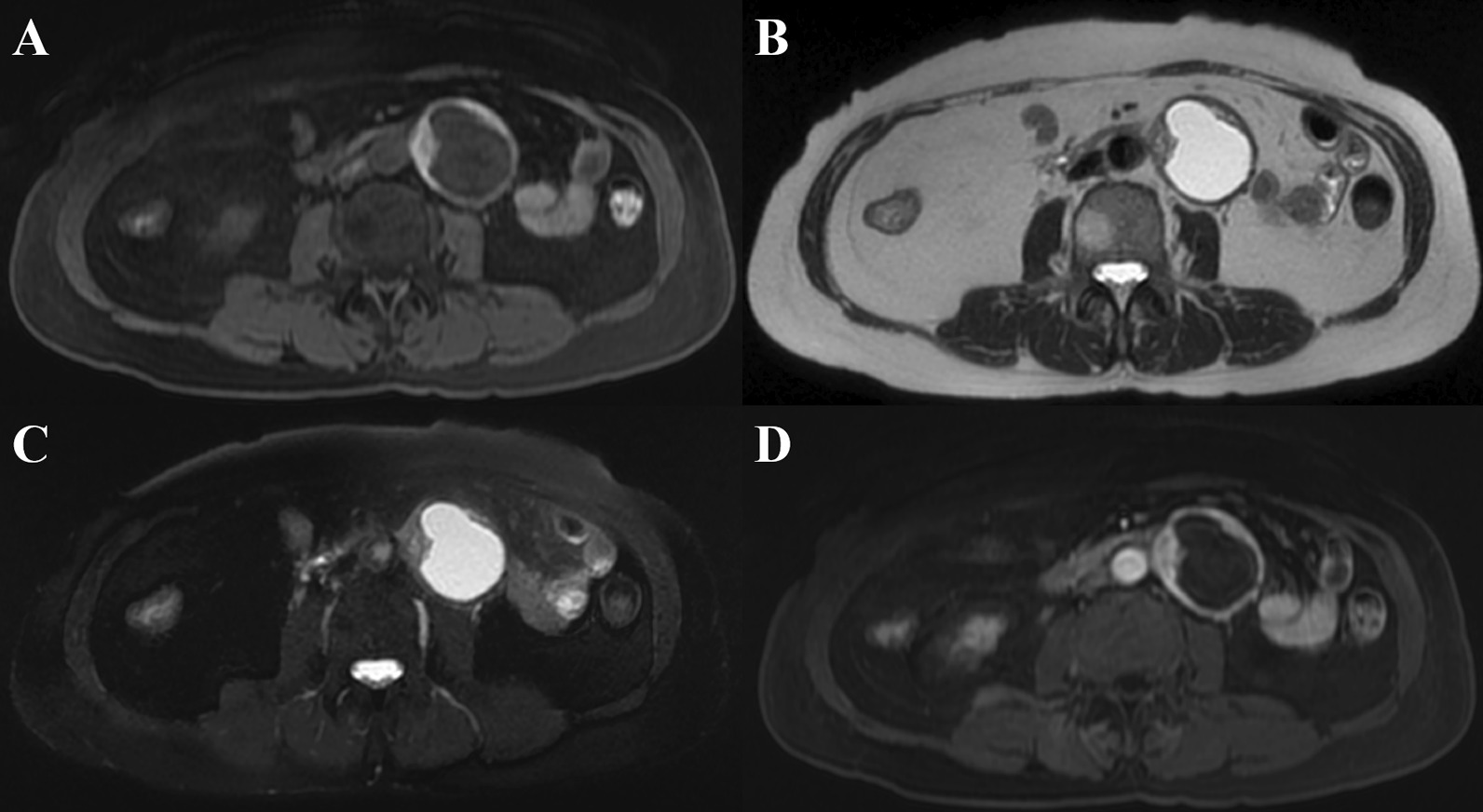
Contrast-enhanced axial MRI scans in a 59-year-old woman with MMNST. **A** A 4.7 cm cystic mass at the para-aortic region, abutting onto the 3rd and 4th portion of the duodenum, showed a hyperintense solid part and a hypointense cystic part on T1WI, and **B** a hypointense solid part and a hyperintense cystic part on T2WI. **C** T2WI with fat suppression also showed a hypointense solid part and a hyperintense cystic part. **D** Contrast-enhanced T1WI showed identical enhancement with T1WI

**Fig. 2:**
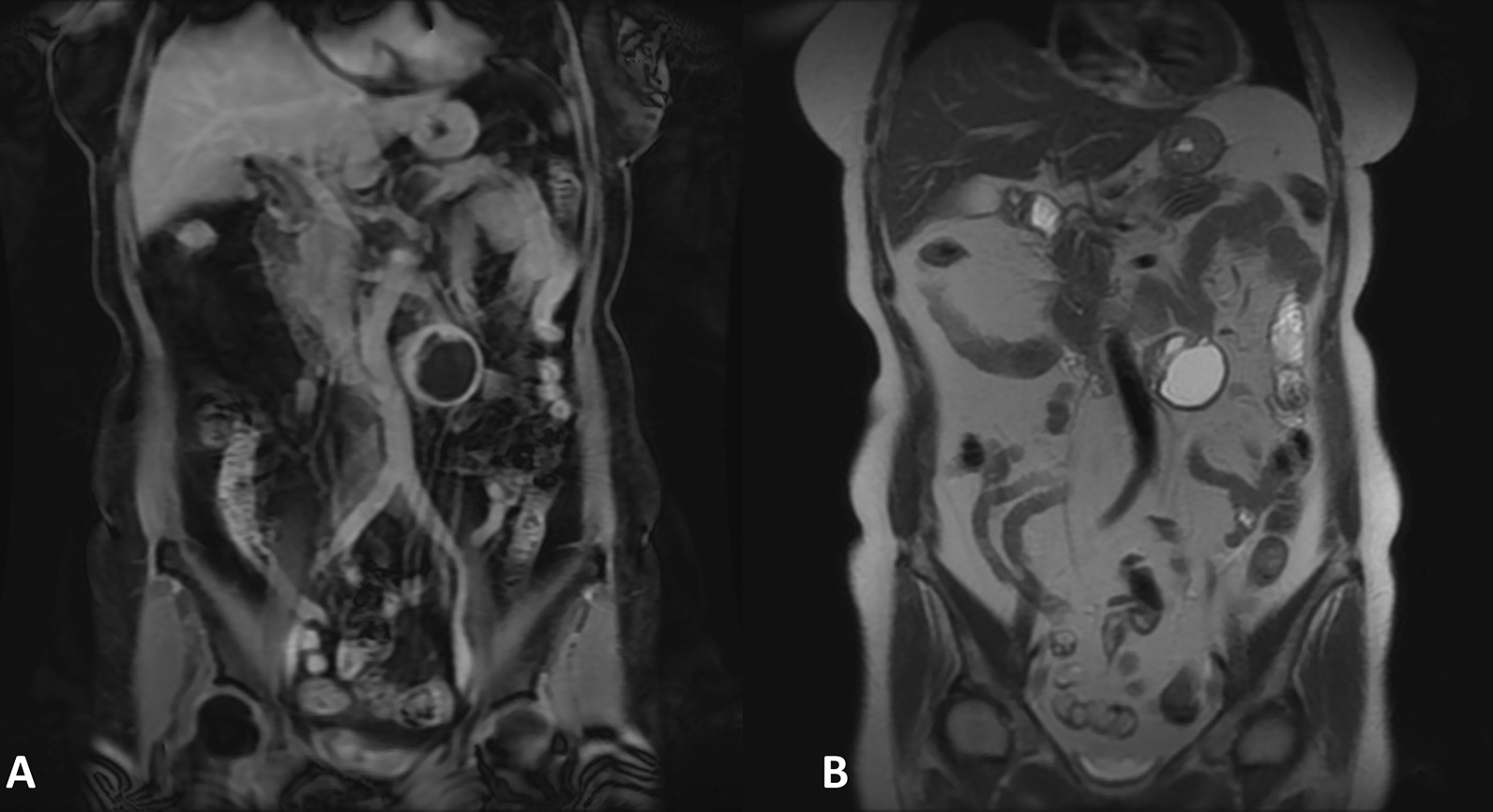
**A** Coronal FIESTA (Fast Imaging Employing Steady-state Acquisition) sequence revealed a 4.7 cm cystic mass with a hyperintense solid part and a hypointense cystic part at para-aortic region, abutting onto the 3rd and 4th portion of the duodenum. **B** Coronal T2WI showed a hypointense solid part and a hyperintense cystic part

Non-contrast CT disclosed a heterogeneous ovoid mass about 52 mm × 41 mm in size. Magnetic resonance imaging (MRI) further revealed a 4.7 cm cystic mass at the para-aortic region abutting to the 3rd and 4th portion of the duodenum (Figs. 1A, 2A). The solid part of the tumor showed hyperintensity on T1WI images and hypointensity on T2WI images (Figs. 1B, 2B). A slightly progressive enhancement was seen in the diffusely restricted portion of the solid part. The apparent diffusion coefficient value was about 1.06 × 10^−3^ mm^2^/s. The cystic part showed hypointensity on T1WI images (Fig. 1A) and hyperintensity on T2WI images (Figs. 1B, 2B), with or without fat suppression (Fig. 1C), and showed identical enhancement in contrast-enhanced T1WI (Fig. 1D). The blood supply to the cystic mass originated from the inferior mesenteric artery (IMA) territory. A gastrointestinal stromal tumor (GIST) was initially diagnosed following differential diagnosis that included paraganglioma.

Her 24-h urine vanillyl mandelic acid and catecholamine values were within the normal range. A paraganglioma was less suspected because her hypertension was less responsive to alpha-blockers.

Laparoscopic surgery was carried out with the patient in a supine position under general anesthesia in April 2021. The tumor was located between the mesocolon and Gerota’s fascia, with a feeding vessel extending from the IMA. The feeding vessel was ligated without compromising the main trunk of the IMA. The tumor was resected en bloc, and a drainage tube was placed. We discharged the patient on postoperative day 4.

**Fig. 3:**
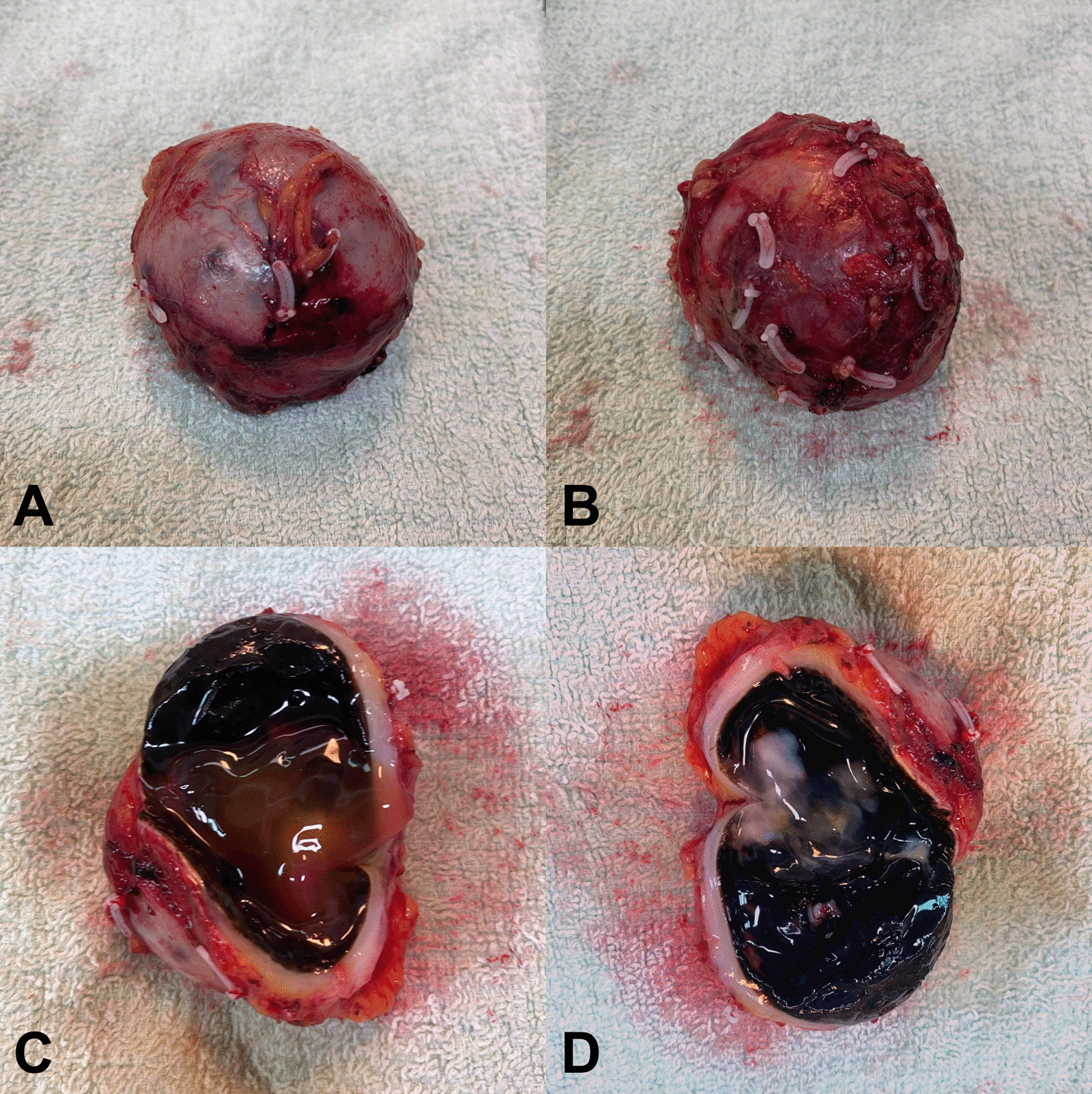
**A**–**D** Representative gross picture of malignant melanocytic nerve sheath tumor (MMNST). The tumor was a well-encapsulated, black, and elastic fibrotic tumor that grossly mimicked melanoma

**Fig. 4:**
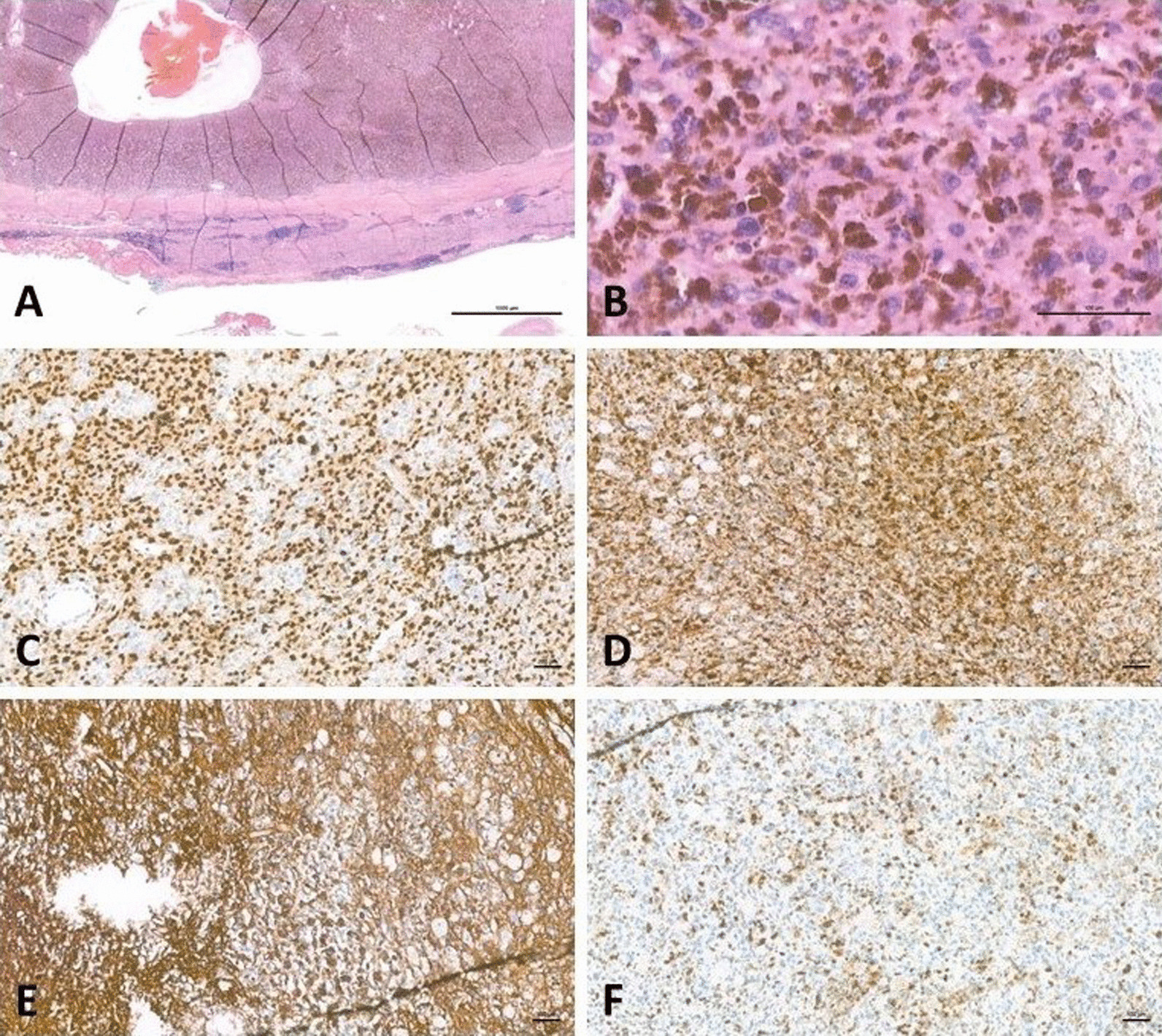
**A** Microscopy revealed a well-encapsulated tumor with lymphoid cuffing; the tumor was composed of pigmented tumor cells and showed cystic degeneration (H&E stain, objective lenses ×4, original magnification ×40, scale bar 1000 μm). **B** Higher magnification revealed that the tumor cells had an epithelioid to spindle shape, mild nuclear atypia, small nucleoli, and abundant intracytoplasmic melanin pigments (H&E stain, objective lenses ×40, original magnification ×400, scale bar 100 μm). **C** Immunohistochemically, the tumor cells were positive for SOX10 (Objective lenses ×10, original magnification ×100, scale bar 100 μm). **D** Immunohistochemically, the tumor cells were positive for HMB45 (Objective lenses ×10, original magnification ×100, scale bar 100 μm). **E** Immunohistochemically, the tumor cells were positive for collagen type IV with a peri-tumoral circumferential pattern (Objective lenses ×10, original magnification ×100, scale bar 100 μm). **F** Immunohistochemically, the tumor cells were negative for AE1/AE3 (Objective lenses ×10, original magnification ×100, scale bar 100 μm). To acquire microscopic images, Nikon Eclipse Ni microscope, Nikon Plan Fluor series lenses, Nikon DS-Ri2 camera, and the acquisition software of NIS-Elements. 5.11.0 were used

Grossly, the specimen was a cystic tissue, 5.8 × 4 × 3.1 cm, with a smooth and partially dark-pigmented outer surface. Dark brown materials were noted at the inner surface of the cystic wall (Fig. 3A–D). Microscopy sections showed an encapsulated tumor with peripheral lymphoid cuffing and intratumoral lymphocytic infiltration (Fig. 4A). The tumor cells were melanin-containing spindled to epithelioid cells featuring pleomorphic and hyperchromatic nuclei and frequent nuclear pseudoinclusions (Fig. 4B). No psammomatous bodies were present. Mitotic activity was as high as 2 per 10 high powered fields (HPFs; 1 HPF × 400 = 0.1734 mm^2^). The pigment was positive for melanin staining and negative for iron staining. Immunohistochemically, the tumor showed positivity for MART1, HMB45, collagen IV, and SOX10, and negativity for AE1/AE3 (Fig. 4C–F). A neurocristic lineage and melanocytic differentiation were noted.

Malignant melanoma should be first excluded because of its poor prognosis. MMNST was favored over malignant melanoma in this case, since the tumor was located near ganglia and had cells with less atypical cytology and a low mitotic rate. The patient also did not present with a history of melanoma. The patient was given a final diagnosis of MMNST. Subsequent adjuvant radiotherapy was completed in July 2021. The patient was alive with no evidence of recurrent or metastatic disease 11 months after radiotherapy.

## Discussion and conclusions

Intra-abdominal MMNST is a rare entity. We reviewed the cases of MMNST in the abdominal cavity without nerve root invasion available in the PubMed database. The features are summarized in Table 1.

**Table 1:** Clinicopathological features of reported intra-abdominal malignant melanotic nerve sheath tumor.

Ref. number	Age	Sex	Site	Size (cm)	Cystic or necrosis	Symptom	Treatment	Follow-up/outcome
1[7]	57	M	Retro, Rt upper	17	+	Urethrorrhagia	GTR	No report
2[8]	40	F	Intercostal near liver	3.9	−	Rt Abd pain	GTR; R/T & C/T after mets	3 yrs/mets and died
3[9]	42	M	Near Rt adrenal	3.8	−	No	GTR	30 mons well
4[10]	16	F	Mesosigmoid	19	−	Abd discomfort	GTR	3 yrs well
5[2]	46	F	Para-aortic	3	N/A	N/A	GTR	60 mons well
6[11]	59	M	Rt renal	15	+	No	GTR	12 mons well
7[12]	43	F	Colon polyp *2	0.8 and 0.5	−	No	Endoscopic remove	No report
8[13]	69	F	Gastric	4.9	−	Nausea, vomit, Ab pain	GTR	3 yrs well
9[14]	67	F	Pancreatic head	5	+	No	GTR	43 mons well
10[15]	77	F	Rt rectus abdominis muscle	4	+	No (except for polymyalgia rheumatica)	GTR	1.5 yrs well
11[16]	67	M	Lt pararenal	12	+	No	GTR	8 mons well
12[17]	73	M	Rt posterior pararenal	5.6	−	Urine frequency + vague Abd pain	GTR	No report
13[18]	75	F	Pancreatic head	7	N/A	Abd pain, vomit, diarrhea	refuse	7 mons well
14[19]	36	F	Behind the liver, Rt paravertebral	11	+	Rt infrascapular pain radiating to her Rt subcostal region	GTR; C/T after recurrence	18 mons, local recurrence
15[20]	51	F	Gastric antrum	19	+	Heartburn and early satiety	GTR	22 mons well
16 present case	59	F	Mesocolon	5.8	+	No	GTR; R/T	11 mons well
17[21]	N/A	N/A	Great omentum	N/A	N/A	N/A	N/A	N/A
18[21]	N/A	N/A	Mesentery	N/A	N/A	N/A	N/A	N/A

*M* male; *F* female; *Rt* right; *Lt* left; *Abd* abdominal; *N/A* not available; *GTR* gross total resection; *mets* metastasis; *R/T* radiotherapy; *C/T* chemotherapy; *mons* months; *yrs* years

Sex was available in 16 cases; 5 of the 16 were male (31.25%) and 11 were female (68.75%). Torres-Mora et al. reported 18 male and 22 female patients in 40 cases of all locations, whereas other previous studies reported no sex predilection [2, 22, 23]. The tumor in the 16 cases had an average maximum diameter of 8.55 cm, with a median of 5.7 cm, compared to a previous study reporting a median tumor size of 3.2 cm [2].

We also assessed the cystic or necrotic changes that occurred in the abdominal MMNSTs. Overall, 14 cases provided structural descriptions, and cystic or necrotic changes were mostly observed in larger sized tumors (≥ 5 cm, 7/9, 77.7%) but less frequently noted in the smaller sized tumors (< 5 cm, 1/5, 20%). All patients underwent surgical removal of the tumor, and no cases had incomplete surgical resection. Follow-up was available in 12 cases, ranging from 7 months to 60 months. The recurrence or metastatic rate were both reported for 1/12 (8%) of these patients and revealed a relatively lower rate than in recent studies [2, 24, 25]. The only patient with metastasis died despite chemotherapy and radiotherapy [8]. Another patient with local recurrence received chemotherapy, but no further follow-up information was provided [19].

MMNST, as updated and defined by the current fifth edition of the WHO classification of tumors of soft tissue and bone [1], was formerly called melanotic schwannoma or melanocytic schwannoma [1]. It is a rare tumor of neural crest derivation and is composed of variably melanin-producing Schwann cells. Fewer than 400 cases have been reported. An MMNST can be located anywhere in the peripheral nerve system and most frequently arises from the region of spinal or autonomic nerves near the midline [1, 26]. The clinical features are related to the anatomic sites and growth. Mostly associated with a mass compression effect, the clinical features could present as pain or paresthesia, or the patients are asymptomatic [1, 26].

The etiology of MMNST is still unknown. Theories have included melanomatous transformation of neoplastic Schwann cells, phagocytosis of melanin by Schwann cells, and the presence of two different neoplastic populations of proliferating melanocytes and Schwann cells [22]. An association is recognized between MMNST and Carney’s complex, an autosomal dominant inherited multiple endocrine neoplastic syndrome mostly caused by a *PRKAR1A* gene deficit [27]. However, the degree of relevance remains controversial, as the presentation ranges from less than 5% to over 50% [2, 4].

Abdominal MMNST often presents with nonspecific abdominal symptoms or is discovered as an incidental finding. GISTs are the most common mesenchymal tumors [19]; therefore, GISTs and MMNSTs should be distinguished. CT scan findings indicate that GISTs seldom show calcification, but calcification may be seen in some MMNSTs [7–9, 28]. MRI findings typically show MMNST as having T1 hyperintensity and T2 hypointensity due to the presence of melanin contained in MMNST. By contrast, GISTs mostly present with opposite findings [9, 24, 29]. These differences in imaging findings may suggest that imaging is a preferable method for differential diagnosis between these two tumors; however, the final diagnosis depends on the histological findings.

Grossly, MMNST presents as a dark brown or black tumor, and it sometimes presents with hemorrhagic components, cyst formation, or necrosis [2]. The tumor is usually ovoid and surrounded by a thin, fibrous membrane that arises related to a nerve [26]. Microscopically, the morphology of MMNST includes spindle and epithelioid cells. Accumulation of melanin occurs in neoplastic cells and melanophages, with variations between different cells [2]. Psammomatous bodies may present occasionally and are more common in Carney’s complex-associated MMNST [6]. Immunohistochemical staining is often positive for S100, SOX10, HMB-45, Melan-A, p16, and vimentin in MMNST [25]. All reported cases of MMNST showed immunoreactions of laminin and collagen IV [30].

Genetically, mutation of the *PRKAR1A* gene is seen in most MMNSTs [31]. The presentation of melanotic features means that malignant melanoma is an important differential diagnosis from MMNST. Tumors with psammomatous bodies, adipose-like cells, benign or mild atypia, and lower mitotic activity would favor a diagnosis of MMNST. In addition, 90% of malignant melanomas present with *BRAF* V600E. Negativity for this test would also indicate MMNST [32, 33].

Gross total excision is widely accepted as the primary treatment for MMNSTs. The local recurrence rate and metastatic rate remain controversial, with < 15% stated in the past and 42% reported in more recent studies [2, 4]. MMNSTs can metastasize even without any malignant features [34]. Adjuvant radiotherapy or chemotherapy have been suggested when malignant features are presented or following incomplete surgical resection, but the effectiveness remains controversial. One study suggested that MMNST with > 2 mitosis/10 HPF should receive adjuvant therapy [34]. Some recent studies have shown an effect of anti-PD1 therapy and the use of Rexin-G® [35, 36]. Follow-up of patients for more than 5 years revealed that only 53% of patients were free of disease, suggesting the importance of long-term follow-up [5].

In this study, we reported an incidentally found intra-abdominal MMNST and reviewed the features of this entity in a total of 18 cases. This study had some limitations. MMNST is an extremely rare entity; therefore, the small sample size, inconsistent treatment protocols, and incomplete follow-up restricted the results of our study. Further evaluation of long-term follow-up and treatment are needed.

Intra-abdominal MMNST is a rare entity. We presented a case with an initial diagnosis of GIST that was subsequently identified as an MMNST located at the para-aortic area. Our review of abdominal MMNST cases showed a female predominance, with an average age of 54.8 years, and a trend toward being a larger tumor showing cystic or necrotic changes. Local recurrence and metastasis rate were reviewed, and both showed a low rate. Diagnosis of MMNST should combine all the available findings, and complete excision of the tumor should be performed, followed by long-term patient monitoring.

## Data Availability

The datasets used and/or analysed during the current study are available from the corresponding author on reasonable request.

## Abbreviations

MMNST: Malignant melanotic nerve sheath tumor
GIST: Gastrointestinal stromal tumor
CT: Computed tomography
MRI: Magnetic resonance imaging
IMA: Inferior mesenteric artery
M: Male
F: Female
Rt: Right
Lt: Left
Abd: Abdominal
N/A: Not available
GTR: Gross total resection
mets: Metastasis
R/T: Radiotherapy
C/T: Chemotherapy
mons: Months
yrs: Years
